# Association of retinal thickness and microvasculature with cognitive performance and brain volumes in elderly adults

**DOI:** 10.3389/fnagi.2022.1010548

**Published:** 2022-11-16

**Authors:** Ruilin Wang, William Robert Kwapong, Wendan Tao, Le Cao, Chen Ye, Junfeng Liu, Shuting Zhang, Bo Wu

**Affiliations:** ^1^Department of Ophthalmology, West China Hospital, Sichuan University, Chengdu, China; ^2^Department of Neurology, West China Hospital, Sichuan University, Chengdu, China

**Keywords:** retinal thickness, retinal microvasculature, cognition, magnetic resonance imaging, optical coherence tomography

## Abstract

**Background:**

Retinal structural and microvascular changes can be visualized and have been linked with cognitive decline and brain changes in cerebral age-related disorders. We investigated the association between retinal structural and microvascular changes with cognitive performance and brain volumes in elderly adults.

**Materials and methods:**

All participants underwent magnetic resonance imaging (MRI), and a battery of neuropsychological examinations. Macula retinal thicknesses (retinal nerve fiber layer, mRNFL, and ganglion cell-inner plexiform layer, GCIPL) were imaged and measured with swept-source optical coherence tomography (SS-OCT) while Optical Coherence Tomography Angiography (OCTA) imaged and measured the superficial vascular complex (SVC) and deep vascular complex (DVC) of the retina.

**Results:**

Out of the 135 participants, 91 (67.41%) were females and none had dementia. After adjusting for risk factors, Shape Trail Test (STT)-A correlated with SVC (*P* < 0.001), DVC (*P* = 0.015) and mRNFL (*P* = 0.013) while STT-B correlated with SVC (*P* = 0.020) and GCIPL (*P* = 0.015). mRNFL thickness correlated with Montreal Cognitive Assessment (MoCA) (*P* = 0.007) and Stroop A (*P* = 0.030). After adjusting for risk factors and total intracranial volume, SVC correlated with hippocampal volume (*P* < 0.001). Hippocampal volume correlated (*P* < 0.05) with most cognitive measures. Stroop B (*P* < 0.001) and Stroop C (*P* = 0.020) correlated with white matter volume while Stroop measures and STT-A correlated with gray matter volume (*P* < 0.05).

**Conclusion:**

Our findings suggest that the retinal structure and microvasculature can be useful pointers for cognitive performance, giving a choice for early discovery of decline in cognition and potential early treatment.

## Introduction

As life expectancies across the world continue to increase, there is predicted to be a rise in the global burden of age-related cognitive impairment. The economic impact of cognitive impairment across the world is tremendous (Pitkala et al., 2021). Individuals with cognitive impairment use more health care services and require greater support with daily living activities (Reppermund et al., 2013); besides individuals with cognitive impairment are at a greater risk of having anxiety, depression, and a lower quality of life (Nys et al., 2006; Hussenoeder et al., 2020). Reducing the incidence or development of cognitive impairment in the aging population is a key target for clinical trials of treatment or intervention for dementia.

Because the retina is suggested as a window to the brain, it is possible to examine early neurodegeneration and microvascular impairment in the central nervous system (CNS) (London et al., 2013). Using different ophthalmic imaging tools, studies have shown patients with dementia and mild cognitive impairment (MCI) have significantly thinner peripapillary retinal nerve fiber layer (pRNFL), macular retinal nerve fiber layer (mRNFL), and ganglion cell-inner plexiform layer (GCIPL) and/or ganglion cell complex (GCC) and microvascular impairment compared to controls without cognitive decline (Alber et al., 2020; Cheung et al., 2021). Structural retinal imaging studies on the elderly population showed thinner RNFL thicknesses and retinal microvascular impairment could indicate cognitive impairment and future cognitive decline over time ultimately resulting in an increased risk of dementia (Ko et al., 2018; Kim et al., 2022). Previous reports reported on either the retinal structure or the retinal microvasculature and its association with cognitive measures (either Montreal Cognitive Assessment, MoCA, or Mini-Mental State Examination, MMSE) in the elderly population.

Our current study aimed to explore the association between the retina (structure and microvasculature), varying array of cognitive measures, and neuroimaging parameters in healthy elderly adults without clinical dementia.

## Materials and methods

The Ethics Committee of West China Hospital of Sichuan University, China, approved this study (2020-104); the protocol of this study adhered to the tenets of the Declaration of Helsinki. Written informed consent was obtained from all participants before enrolling in this study.

### Participants

We enrolled Chinese individuals who were 50 years or older from Chengdu, Sichuan, China as part of a healthy aging study from the Neurology Department of West China Hospital. Inclusion criteria included neurologic and neuropsychological examination with normative standards as previously reported (Casaletto et al., 2017); no major memory concerns or a diagnosed memory disorder; the capability to independently complete activities of daily living with a clinical dementia rating of 0 (Duff et al., 2022).

Participants answered questionnaires covering demographic, education, and self-reported vascular risk factors including hypertension, diabetes, smoking, and alcohol consumption information.

### Neuropsychological assessment

All participants enrolled in our study underwent Shape Trail Test (STT), Chinese Rey Auditory Verbal Learning Test (C-RAVLT), and Stroop Color and Word Test (SCWT). The STT is a variant form of the classic Trail Making Test (TMT) which is widely used in China (Zhao Q. et al., 2013; Yang et al., 2021). For STT, patients underwent STT-A (drawing a line between 25 consecutive numbers as fast as possible) and STT-B (linking numbers alternating between circles and squares). SCWT consists of three cards printed in color as previously reported (Stroop, 1992; Zysset et al., 2001). It is widely used to evaluate the basic human executive function, particularly attention and information processes. The Dot subtask (card A) consists of color dots, the word subtask (card B) consists of common words unrelated to the concept of colors, and the Color-Word subtask (card C) consists of words that are names of colors. The colors used are blue, green, red, and yellow. Participants were required to name the colors in which the stimuli were printed and to disregard their verbal content. For the Chinese version (CST) of the SCWT, common Chinese characters unrelated to the concept of color were selected. Completion times and correct numbers were recorded. The Chinese Rey Auditory Verbal Learning Test (C-RAVLT) was developed to measure verbal learning and memory domain. The test consists of five successive presentations of the original list of 15 words (List A), with each trial followed by a free recall. The total number of correctly remembered words in all 5 trials was taken as the score for immediate recall (IR). Participants were required to recall words from List A again after a 30-min delay as long-delay recall (DR). Mini-Mental State Examination (MMSE) and Montreal Cognitive Assessment (MoCA-BJ) (Yu et al., 2012) were also assessed for all participants. All tests were performed by a professionally trained physician.

### Brain image acquisition and volumetric measures of brain structure

Image acquisition was performed using a standard 3T scanner (Siemens Skyra) with a 32- channel head coil at West China Hospital of Sichuan University. Sequences consisted of T1 and T2-weighted imaging, fluid-attenuated inversion recovery (FLAIR), and susceptibility-weighted imaging (SWI). T1-weighted high-resolution images were acquired by a 3D magnetization-prepared rapid gradient echo (MPRAGE). Imaging parameters were TR = 1,900 ms; TE = 2.4 ms; FA = 9°; FOV = 250 mm; 256 × 192 matrix; 191 slices; voxel dimension = 1.0 mm × 1.0 mm × 1.0 mm.

Computational Anatomy Toolbox 12 (CAT12)^1^ was used to process T1-weighted structural images for Statistical Parametric Mapping (SPM) 12 (Wellcome Trust Center for Neuroimaging, London, UK). First, each structural image was visually inspected for artifacts and then reoriented to set the image origin at the anterior commissure. Secondly, the reoriented images were spatially normalized to Montreal Neurological Institute space and segmented into gray matter (GM), white matter (WM), and cerebrospinal fluid, using the standard tissue probability maps provided in SPM12. Then, Jacobian modulation was adjusted using volume changes induced by normalization. With modulation, voxel-based Morphometry (VBM) was considered as comparing the absolute volume of gray or white matter structures, by multiplying the spatially normalized structure by its relative volume before and after spatial normalization. Spatially normalized GM images were finally smoothed using a Gaussian kernel with a full width at half a maximum of 8 mm. Total intracranial volumes (TIVs) were calculated by summing the volume values of the GM, WM, and cerebrospinal fluid. Bilateral hippocampus volumes were calculated using the automated anatomical labeling (AAL) template.

### Swept-source optical coherence tomography and swept-source optical coherence tomography angiography examination

**FIGURE 1:**
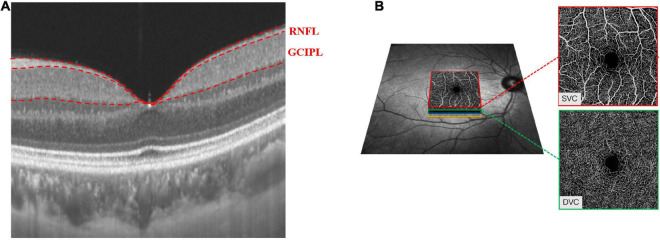
Retinal microvasculature and sub-layer thicknesses in a 3 mm × 3 mm area around the fovea. The radial scan model shows the RNFL and GCIPL thicknesses of the macula **(A)**. The SVC and DVC were set in the inner two-thirds and outer one-third border of GCIPL **(B)**.

SS-OCT/SS-OCTA (VG200S; SVision Imaging, Henan, China; version 2.1.016) was used to image the retinal structure and microvasculature of all participants. Retinal image acquisition was done in both eyes. Previous reports have detailed the specifications of the SS-OCT/OCTA tool (Kwapong et al., 2022a,b; Tao et al., 2022; Ye et al., 2022). Structural OCT imaging was done with 18 radial B-scans positioned on the fovea. Each B-scan was generated by 2048 A-scans, was 12 mm long, and separated from adjacent lines by 10 degrees. Each B-scan was automatically averaged 64 times to improve the signal-to-noise ratio (Alonso-Caneiro et al., 2011). Automatic segmentation of the retinal thickness was done by the OCT tool. Our current study focused on the macular retinal nerve fiber layer (mRNFL), and ganglion cell-inner plexiform layer (GCIPL) in a 3 × 3 mm^2^ area around the fovea as shown in Figure 1A. The OCT tool provided the mean thicknesses (measured in μm) of the retinal structure.

The OCTA images covered an area of 3 × 3 mm^2^ centered on the fovea. The *en face* angiograms of the superficial vascular complex (SVC) and deep vascular complex (DVC) were generated by the OCTA tool. The segmentation of the SVC and DVC slabs was set in the inner two-thirds and outer one-third border of GCIPL as shown in Figure 1B. Mean percentages (%) of the microvasculature in the SVC and DVC were obtained with an in-built algorithm in the OCTA tool.

All retinal measurements were done at the macula. OCT/OCTA data displayed in our study followed the OSCAR-IB quality criteria (Tewarie et al., 2012) and APOSTEL recommendation (Aytulun et al., 2021).

### Statistical analysis

Continuous variables with normal distribution were expressed as mean ± standard deviation (SD), while skewed distribution was shown as medians and interquartile ranges. Categorical variables are presented as frequencies and percentages (%). The z scores of all brain MRI volumes and cognitive measures were calculated. Multivariate linear regression based on a generalized estimating equation was used to investigate the association between SS-OCT/OCTA parameters, cognitive measures, and neuroimaging parameters while adjusting for gender, age, inter-eye dependencies, and vascular risk (hypertension, diabetes mellitus, hyperlipidemia, alcohol intake, and current smokers). We additionally adjusted for education years when the outcome was the cognitive measures and hippocampal volume. Multivariate regression was used to investigate the association between neuroimaging parameters and cognitive function while adjusting for risk factors and intracranial volume. All analyses were performed with R version 4.2.1 using R Studio and R Markdown (RStudio Inc., Boston, MA). *P*-values less than 0.05 (*P* < 0.05) were considered statistically significant. The Bonferroni method was used for the correction of multiple comparisons. We performed a *post-hoc* power calculation to evaluate the statistical power of GEE in our study; the statistical power of GEE in our study was *P* < 0.001.

## Results

**FIGURE 2:**
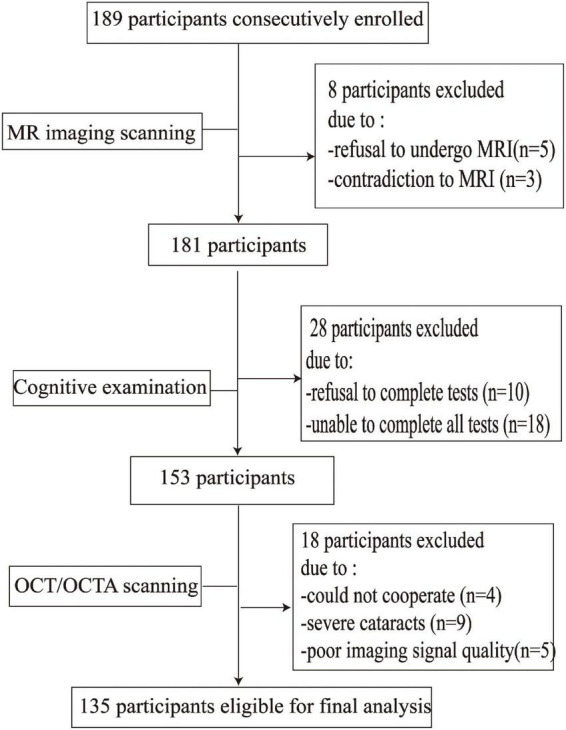
Flow chart diagram of the inclusion and exclusion criteria of our participants.

Figure 2 shows the flow chart of our data cohort. Two hundred and sixty-four eyes from 135 participants were included in our data analysis; 6 eyes were excluded because of poor imaging quality. The characteristics of the analyzed data are shown in Table 1. The median age was 58 years (IQR 53–64 years) and the median years of education were 12. Out of the 135 participants, 91 (67.41%) were females and none had dementia; 24 had a history of hypertension while 21 (17.04%) were current smokers and 28 (20.74%) were current drinkers. The average SS-OCT/OCTA parameters were as follows: mRNFL thickness = 18.62 ± 1.77 μm, GCIPL thickness = 69.65 ± 7.04 μm, SVC density = 35.86 ± 5.14%, and DVC density = 46.36 ± 4.54% as shown in Table 1.

**TABLE 1:** Characteristics of the study population.

Characteristics	*N*
Number	135
Age (years)	58 (53–64)
Gender (females)	91 (67.41%)
Education (years)	12 (9–16)
Hypertension (*n*)	24 (17.78%)
Diabetes (*n*)	4 (2.96%)
Dyslipidemia (*n*)	23 (17.04%)
Smokers (*n*)	21 (15.56%)
Drinkers (*n*)	28 (20.74%)
**Cognitive function, raw scores**	
MMSE	29 (28–30)
MoCA	26 (24–28)
Stroop A (s)	27 (22–31)
Stroop B (s)	41 (35–53)
Stroop C (s)	75 (63–96)
STT-A (s)	22 (16–36)
STT-B (s)	138 (111–192)
Total intracranial volume	1,360.71 (1,294.45–1,475.27)
Average hippocampal volume	3.98 (3.73–4.93)
GMV	602 (573.49–630.94)
WMV	484.06 (461.02–523.53)
SVC (%)	35.86 ± 5.14
DVC (%)	46.36 ± 4.54
RNFL (μm)	18.62 ± 1.77
GCIPL (μm)	69.65 ± 7.04

MMSE, Mini-Mental State Examination; MoCA, Montreal Cognitive Assessment; STT, Shape Trail Test; GMV, gray matter volume; WMV, white matter volume; SVC, superficial vascular complex; DVC, deep vascular complex; RNFL, retinal nerve fiber layer; GCIPL, ganglion cell-inner plexiform layer.

**FIGURE 3:**
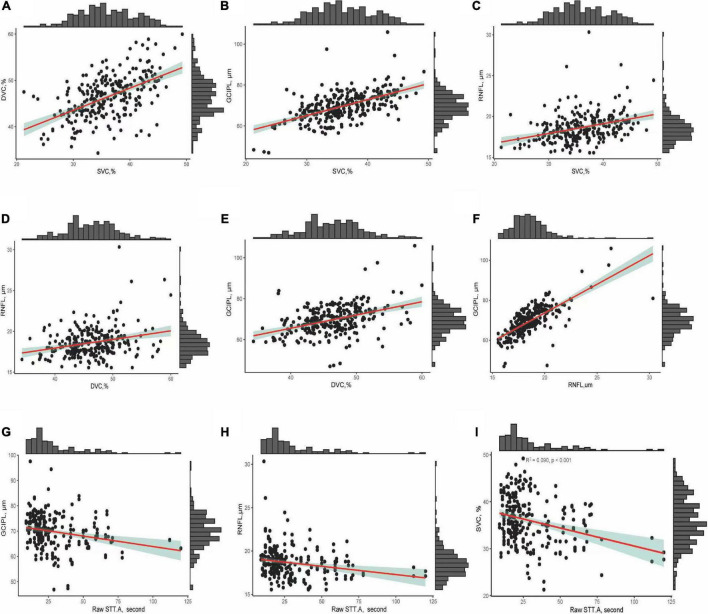
**(A–F)** Show the correlation between OCT/OCTA parameters. **(G–I)** Show the correlation between OCT/OCTA parameters and STT-A without adjusting for risk factors.

Figure 3 shows the correlation between OCT and OCTA parameters and the association between OCT/OCTA parameters and cognitive measures. Retinal thicknesses positively correlated with microvascular densities; importantly, STT-A correlated with mRNFL, GCIPL, and SVC (all *P* < 0.001).

### Correlation between swept-source optical coherence tomography/optical coherence tomography angiography parameters and cognitive measures

Table 2 shows the correlation between OCT/OCTA parameters and cognitive measures after adjusting for risk factors. STT-A correlated with SVC (*P* < 0.001), DVC (*P* = 0.015) and mRNFL (*P* = 0.013) while STT-B correlated with SVC (*P* = 0.020) and GCIPL (*P* = 0.015). mRNFL thickness correlated with MoCA (*P* = 0.007) and Stroop A (*P* = 0.030). Importantly, SVC correlated with (STT B – STT A; *P* < 0.001, data not shown). DVC density (*P* = 0.070), mRNFL (*P* = 0.696), and GCIPL (*P* = 0.846) thicknesses did not show any significant correlation with the difference between STT B and STT A.

**TABLE 2:** Correlation between SS-OCT/OCTA parameters and cognitive measures.

		MMSE	MoCA	Stroop A	Stroop B	Stroop C	STT-A	STT-B	STT B - STT A	RAVLT (IR)	RAVLT DR
SVC	B	0.193	0.088	–0.529	–0.922	–0.396	–1.700	1.016	1.251	0.603	–0.057
	SE	0.477	0.528	0.393	0.427	0.389	0.476	0.433	0.329	0.340	0.355
	*P*-value	0.686	0.867	0.180	0.031*	0.310	< 0.001*	0.020*	< 0.001*	0.077	0.872
DVC	B	–0.252	–0.162	–0.155	–0.688	0.181	–0.965	0.366	0.500	0.172	–0.129
	SE	0.390	0.432	0.323	0.350	0.319	0.394	0.358	0.274	0.280	0.290
	*P*-value	0.518	0.078	0.631	0.051	0.572	0.015*	0.307	0.070	0.539	0.657
RNFL	B	0.133	0.463	–0.346	–0.117	–0.102	–0.347	–0.101	–0.043	–0.043	–0.002
	SE	0.156	0.170	0.158	0.143	0.129	0.139	0.127	0.110	0.112	0.116
	*P*-value	0.393	0.007*	0.030*	0.413	0.429	0.013*	0.429	0.696	0.695	0.984
GCIPL	B	0.299	1.139	–0.242	–1.528	0.363	–0.98	0.320	0.084	–0.850	–0.690
	SE	0.616	0.678	0.566	0.622	0.510	0.553	0.504	0.434	0.438	0.456
	*P*-value	0.628	0.095	0.667	0.015*	0.476	0.078	0.525	0.846	0.054	0.132

*P*-values adjusted for age, gender, education, smoking, drinking, hypertension, diabetes, and dyslipidemia.

B, beta coefficient; SE, standard error; MMSE, Mini-Mental State Examination; MoCA, Montreal Cognitive Assessment; STT, Shape Trail Test; IR, immediate recall; DR, delayed recall; SVC, superficial vascular complex; DVC, deep vascular complex; RNFL, retinal nerve fiber layer; GCIPL, ganglion cell-inner plexiform layer.

*Significant difference (*P* < 0.05).

### Correlation between swept-source optical coherence tomography/optical coherence tomography angiography parameters and neuroimaging parameters

After adjusting for risk factors and total intracranial volume, SVC correlated with average hippocampal volume (*P* < 0.001, Table 3).

**TABLE 3:** Correlations between SS-OCT/OCTA parameters and brain MRI volume.

		Hippocampus*^l^*	WMV	GMV
SVC	B	–1.675	–0.013	–0.019
	SE	0.311	0.012	0.014
	*P*-value	< 0.001*	0.290	0.180
DVC	B	–0.33	–0.01	–0.022
	SE	0.283	0.01	0.126
	*P*-value	0.244	0.343	0.071
RNFL	B	–0.035	0.006	–0.005
	SE	0.107	0.004	0.004
	*P*-value	0.746	0.113	0.290
GCIPL	B	–0.059	0.019	–0.01
	SE	0.423	0.016	0.019
	*P*-value	0.889	0.237	0.612

*P*-values adjusted for age, gender, smoking, drinking, hypertension, diabetes, dyslipidemia, and total intracranial volume.

B, beta coefficient; SE, standard error; SVC, superficial vascular complex; DVC, deep vascular complex; RNFL, retinal nerve fiber layer; GCIPL, ganglion cell-inner plexiform layer, WMV, white matter volume; GMV, gray matter volume.

^*l*^*P*-values adjusted for age, gender, education, smoking, drinking, hypertension, diabetes, dyslipidemia, and total intracranial volume.

*Significant difference (*P* < 0.05).

### Correlation between neuroimaging parameters and cognitive measures

We observed multiple correlations between cognitive measures and neuroimaging parameters as shown in Table 4; the average hippocampal volume correlated (*P* < 0.05) with most cognitive measures. Stroop B (*P* < 0.001) and Stroop C (*P* = 0.020) correlated with WMV while Stroop measures and STT-A correlated with GMV (*P* < 0.05).

**TABLE 4:** Correlation between cognitive measures and brain MRI volume.

	Hippocampus			WMV			GMV		
			
	B	SE	*P*-value	B	SE	*P*-value	B	SE	*P*-value
MMSE	0.209	0.093	0.026*	0.912	2.487	0.715	1.545	2.108	0.464
MoCA	0.28	0.101	0.006*	2.923	2.739	0.285	–4.435	2.293	0.054
Stroop A	0.329	0.074	< 0.001*	–0.239	2.035	0.906	5.552	1.682	0.001*
Stroop B	0.241	0.082	0.003*	–8.734	2.143	< 0.001*	5.425	1.84	0.003*
Stroop C	0.153	0.075	0.043*	–4.672	1.996	0.020*	4.926	1.676	0.004*
STT-A	0.614	0.086	< 0.001*	–0.713	2.512	0.777	8.236	2.057	0.001*
STT-B	–0.088	0.085	0.299	–1.231	2.248	0.585	2.209	1.896	0.245
RAVLT (IR)	–0.218	0.065	0.001*	–1.574	1.767	0.374	–1.163	1.493	0.437
RAVLT (DR)	–0.051	0.069	0.466	0.603	1.84	0.740	–0.308	1.559	0.843

*P*-values adjusted for age, gender, education, smoking, drinking, hypertension, diabetes, dyslipidemia, and total intracranial volume.

B, beta coefficient; SE, standard error; MMSE, Mini-Mental State Examination; MoCA, Montreal Cognitive Assessment; STT, Shape Trail Test; I C-RAVLT (IR), The Chinese Rey Auditory Verbal Learning Test (immediate recall); C-RAVLT (DR), The Chinese Rey Auditory Verbal Learning Test (delayed recall); GMV, gray matter volume; WMV, white matter volume.

*Significant difference (*P* < 0.05).

## Discussion

The present study showed that retinal thickness measured by OCT and retinal microvasculature measured by OCTA correlated with cognitive measures. After adjusting for risk factors and total intracranial volume, neuroimaging analysis showed hippocampal volume correlated with SVC density. Similar significant associations were also observed between cognitive measures and neuroimaging parameters. Our findings suggest that retinal thickness and microvasculature may reflect neuroimaging parameters associated with domains of cognition function and ensuing cognitive impairment in elderly adults.

STT is based on Trail Making Test and was developed for individuals who speak Chinese Mandarin as their first language. Population studies (Ko et al., 2018; Mutlu et al., 2018) have shown an association between thinner RNFL with worse cognitive performance (STT-A). We showed STT-A scores inversely correlated with SVC and DVC densities and RNFL thickness. STT-A assesses executive function, specifically attention and cognitive speed (Zhao Q. et al., 2013); these functions are frequently impaired in elderly adults and the aging population with cerebral disorders. OCTA reports have shown patients with dementia and other cerebral disorders have reduced SVC density and thinner RNFL and GCIPL thicknesses compared to controls (Kashani et al., 2021; Snyder et al., 2021). Thus, the associations between SS-OCT/OCTA parameters and STT examination give meaningful suggestions for retinal imaging research for cognitive assessment in elderly individuals. Noteworthy, we showed that SVC correlated with the difference in STT-B and STT-A, which removes the influence of speed.

Stroop is a cognitive tool that assesses the executive function of an individual, primarily focused attention; it helps in evaluating the behavioral control functions using the conflict between perception and speech (Zhao J. H. et al., 2013). Lee et al. suggested that poorer performances on Stroop B and Stroop C reflect genuine cognitive impairment. In our present study, we showed retinal thicknesses and retinal microvasculature had a significant correlation with Stroop parameters which is in line with previous reports (van Koolwijk et al., 2009; Mutlu et al., 2018). Our findings suggest that GCIPL thickness and SVC density may reflect cognitive dysfunction which is in line with previous reports (Jones-Odeh et al., 2016; Ko et al., 2018; Girbardt et al., 2021; Kim et al., 2022).

MoCA, as a general cognitive screening tool, correlated with RNFL thickness in our study. Previous reports (Oktem et al., 2015; Liu et al., 2019; Jeevakumar et al., 2022) have shown a significant correlation between OCT parameters and MoCA scores. Our findings suggest that thinner RNFL may indicate reduced MoCA scores which are in line with previous reports.

The pathophysiological elucidation for the association between SS-OCT/OCTA parameters and cognitive measures is interesting. Of note, these neuropsychological examinations require vision and the retina is the main processing visual medium in the central nervous system. Interpretation of these tests is accomplished in the retina where dementia reports have shown microvascular impairment and neurodegeneration (Snyder et al., 2021). Even though cognitive dysfunction may be related to cerebral damage, the involvement of the retina (both structural and microvessels) in cognition is supported by prior OCT/OCTA reports (Kashani et al., 2021; Snyder et al., 2021).

The underlying mechanism of SVC density and hippocampal volume is vague. It is postulated that retinal microvasculature reflects cerebral microcirculation. Animal reports (Koronyo et al., 2017; Shi et al., 2021) on dementia models showed deposition of amyloid-beta and tau in the retinal vessels and inner retinal structure, and autopsy reports (Blanks et al., 1989, 1996) on humans also showed these pathological changes in the retina. We showed a significant correlation between SVC density and hippocampal volume. Deposition of amyloid-beta, the pathological hallmark of dementia, has been reported to occur in the hippocampus and retina of mice simultaneously (Perez et al., 2009), suggesting that the development of dementia may involve neuron cell death of the brain (hippocampal atrophy) but also microvascular impairment of the retina. Similarly, hippocampal atrophy is a radiology indicator for Alzheimer’s disease or dementia (Woodworth et al., 2022); recent reports suggest hippocampal atrophy may be due to vascular pathology, specifically small vessel disease and ischemia, which are implicated in the development of dementia (Kalaria, 2000; Iadecola, 2013). OCTA reports (Alber et al., 2020; Cheung et al., 2021) have shown reduced SVC density in patients with dementia compared to controls indicating ischemia. Given that hippocampal atrophy is a prominent feature of dementia, our findings that showed SVC density correlated with hippocampal volume suggests that microvascular impairment may lead to hippocampal atrophy.

It is suggested that the hippocampus plays a significant role in memory retrieval (St-Laurent et al., 2016; Ross et al., 2018); thus, hippocampal damage is linked with memory performance. We showed that hippocampal volume correlated with most cognitive measures and immediate recall memory. Our findings suggest that hippocampal volume is associated with cognitive measures and may contribute to recall memory particularly highlighting the role of cognitive speed and executive abilities in addition to information storage capacity (Friedman and Johnson, 2000). We also showed gray matter volume and white matter volume correlated with cognitive measures which are in line with previous reports (Sasson et al., 2013; Ramanoel et al., 2018).

Investigation of the association between age-related changes in regional brain volumes and changes in cognitive measures may provide insights into the neural underpinnings of cognitive aging. Hippocampal volume, white matter, and gray matter volume correlated with cognitive tools which are reflective of executive function. Our findings highlight the clinical importance of volumetric analysis and its association with cognition in the aging population.

Mutlu et al. (2017) showed thinner peripapillary RNFL (pRNFL) thickness (thickness around the optic nerve head, ONH, which reflects the axonal integrity) was associated with smaller white matter volume and gray matter volume. Chua et al. (2021) did not show an association between mRNFL (retinal ganglion cell axon thickness at the macular) and white matter volume; the authors suggested that compared to the pRNFL, mRNFL is thinner and may not be sensitive to neurodegeneration. However, our current study did not show any significant association between retinal thickness at the macular and neuroimaging volumes. Further studies are needed to elucidate the association between retinal thickness and cerebral microstructural volumes.

There are some limitations in our study we would like to acknowledge. The observational, cross-sectional study design of our study is a major limitation, thus the association between SS-OCT/OCTA parameters, cognitive measures, and volumetric analysis of the brain should be interpreted with caution. Secondly, there was a possibility of a selection bias caused by the exclusion of individuals from our final data analysis; we excluded individuals with retinal abnormalities such as age-related macular degeneration (AMD), which are prevalent in aging individuals. This may have led to an underestimation of the observed correlations. Importantly, the participants enrolled in our study were Chinese which limits the generalizability of our findings. Thus, we suggest that the findings of our study should be corroborated by other races. Vision is needed for all neuropsychological examinations and the retina plays a significant role in visual processing; our study did not perform a visual acuity examination on our participants. The strengths of our study include the assessment of cerebral microstructural volume, retinal microvasculature, and structure. Both eyes were included in the analysis and GEE was used to account for the inter-eye correlation of an individual. Another strength of this study is the varying cognitive assessments performed in our study to reflect different cognitive domains. Thus, the association between these cognitive tools and retinal structure and microvasculature suggests that the retina may reflect cognition in elderly adults.

Our present study showed retinal thicknesses and microvasculature significantly correlated with cognitive measures and cerebral volumetric analysis in aging individuals. Our findings suggest that measurement of the retinal thickness and microvasculature by the SS-OCT/OCTA may be useful markers for assessing changes in cognitive function in elderly adults. Taken together, our study suggests that the retina (both structure and microvasculature) can be a pointer for cognitive performance, giving a choice for early discovery of decline in cognition and potential early treatment. Further studies with large sample sizes are required to validate the findings of this study.

## Data availability statement

The original contributions presented in this study are included in the article/supplementary material, further inquiries can be directed to the corresponding authors.

## Ethics statement

The studies involving human participants were reviewed and approved by the Ethics Committee of West China Hospital. The patients/participants provided their written informed consent to participate in this study.

## Author contributions

WK, RW, and WT: study concept, design, and data acquisition. RW, WK, LC, SZ, WT, JL, and CY: data analysis and interpretation. RW, JL, CY, WT, SZ, and WK: drafting of the manuscript. WK, RW, and SZ: critical review of manuscript. All authors approved of this version of the manuscript.

## Footnotes

1
http://dbm.neuro.uni-jena.de/cat/
